# Chicken Pleiotrophin: Regulation of Tissue Specific Expression by Estrogen in the Oviduct and Distinct Expression Pattern in the Ovarian Carcinomas

**DOI:** 10.1371/journal.pone.0034215

**Published:** 2012-04-04

**Authors:** Jin-Young Lee, Wooyoung Jeong, Whasun Lim, Jinyoung Kim, Fuller W. Bazer, Jae Yong Han, Gwonhwa Song

**Affiliations:** 1 WCU Biomodulation Major, Department of Agricultural Biotechnology, Seoul National University, Seoul, Korea; 2 Department of Animal Science, Center for Animal Biotechnology and Genomics, Texas A&M University, College Station, Texas, United States of America; Beckman Research Institute of City of Hope, United States of America

## Abstract

Pleiotrophin (PTN) is a developmentally-regulated growth factor which is widely distributed in various tissues and also detected in many kinds of carcinomas. However, little is known about the *PTN* gene in chickens. In the present study, we found chicken PTN to be highly conserved with respect to mammalian *PTN* genes (91–92.6%) and its mRNA was most abundant in brain, heart and oviduct. This study focused on the *PTN* gene in the oviduct where it was detected in the glandular (GE) and luminal (LE) epithelial cells. Treatment of young chicks with diethylstilbesterol induced PTN mRNA and protein in GE and LE, but not in other cell types of the oviduct. Further, several microRNAs, specifically *miR-499* and *miR-1709* were discovered to influence PTN expression via its 3′-UTR which suggests that post-transcriptional regulation influences PTN expression in chickens. We also compared expression patterns and CpG methylation status of the *PTN* gene in normal and cancerous ovaries from chickens. Our results indicated that PTN is most abundant in the GE of adenocarcinoma of cancerous, but not normal ovaries of hens. Bisulfite sequencing revealed that 30- and 40% of −1311 and −1339 CpG sites are demethylated in ovarian cancer cells, respectively. Collectively, these results indicate that chicken PTN is a novel estrogen-induced gene expressed mainly in the oviductal epithelia implicating PTN regulation of oviduct development and egg formation, and also suggest that PTN is a biomarker for epithelial ovarian carcinoma that could be used for diagnosis and monitoring effects of therapies for the disease.

## Introduction

Pleiotrophin (PTN), also known as heparin-binding growth factor 8 (HBGF-8) and heparin-binding growth associated molecule (HB-GAM), is a low molecular weight protein (about 18 kDa) which was originally isolated from the bovine uterus [1]. As a member of developmentally regulated cytokine/growth factor family, it is widely distributed in various tissues and especially plays pivotal roles in neurogenesis and epithelial mesenchymal interactions through promoting cell growth and migration during early embryo differentiation and morphogenesis [2], [3], [4]. PTN binds to its cognate receptor protein tyrosine phosphatase beta/zeta (RPTP β/ζ) [5] and then activates several cytosolic proteins such as CTNNB1 (beta-catenin), ADD2 (beta-adducin), FYN (Fyn oncogene related to SRC, FGR, YES) and ALK (anaplastic lymphoma receptor tyrosine kinase) for many different cellular functions and systems [4]. In addition, PTN is a proto-oncogene [6] expressed in malignant tumors and cell lines of various organs such as breast, prostate, colon, lung and skin and is thought to be involved in tumor angiogenesis and metastasis [4], [7], [8], [9], [10]. In spite of the fact that PTN is involved in the regulation of cellular development and differentiation, and the etiology of carcinogenesis in many vertebrates, little is known about its expression and regulation by steroid hormones in the oviduct or its expression in normal and cancerous ovaries of chickens.

The chicken oviduct is one of best animal models for studies of organ development and differentiation, and biological actions and signaling pathways for steroid hormones such as estrogen [11]. As a representative sex hormone in female reproductive organs, estrogen not only regulates reproductive behavior but also stimulates epithelial cells within the immature oviduct to transform into tubular gland cells via proliferation and cytodifferentiation, as well as transactivation of oviduct-specific genes such as ovalbumin during development of the chicken oviduct [12], [13]. In addition, estrogen plays a pivotal role in calcium metabolism and calicification of the eggshell prior to oviposition in laying hens [14], [15], and exogenous estrogen administration to postnatal chicks induces cellular hyperplasia and hypertrophy of the oviduct resulting in its rapid growth rate and maturation [11], [16].

The laying hen is a unique animal model for research on human ovarian cancer because it spontaneously develops epithelial cell-derived ovarian cancer as in women [17], [18], [19], [20], [21]. Indeed, ovarian cancer is the most lethal gynecological disease as well as the 5^th^ leading cause of cancer-derived deaths among women in the U.S.A. [22], [23], [24], because it is rarely diagnosed at an early stage due to the lack of a specific biomarker(s) for early detection and it is generally asymptomatic [17], [25], [26]. Among three types of ovarian cancers, i.e., epithelia-, germ cell-and stroma-derived malignant tumors [27], [28], germinal epithelia-derived ovarian cancer (EOC) accounts for over 90% of ovarian cancer incidences in women [29]. This high rate of EOC incidence likely results from incessant ovulation and menstrual cycles that lead to genomic damage and mutations in genes in the ovarian surface epithelium [30], [31]. To investigate and elucidate the etiological and pathological aspects of EOC, several rodent models have been developed through biotechnological manipulation, but they have many limitations and obstacle associated with clinical relevance because of the non-spontaneous nature and physiologically distinct differences in their EOC [17], [32], [33]. Meanwhile, the chicken spontaneously develops EOC at a high rate as occurs in women and shows very similar morphological characteristics to that of EOC in women [17], [18], [19], [20], [21]. Therefore, the chicken EOC could be used to develop anti-cancer drugs and biomarkers for early diagnosis and therapies to prevent adverse outcomes of EOC in women.

We reported that the avian homolog of the human *PTN* transcript is highly expressed in chicks treated with the synthetic estrogen agonist diethylstilbestrol (DES) [34]. However, little is known about the expression and function of PTN in most species except humans and mice [35]. Therefore, the objectives of the present study were to: 1) investigate tissue- and cell-specific expression of the *PTN* gene in chickens; 2) determine whether estrogen regulates expression of *PTN* during oviduct development in chicks; 3) determine whether *PTN* is regulated by post-transcriptional actions of specific microRNAs; 4) compare differential expression of *PTN* in normal and cancerous ovaries from hens and 5) examine CpG methylation status in the upstream promoter region of the *PTN* gene in normal and cancerous ovarian cells from hens. Results of current study indicate that *PTN* is a novel estrogen-stimulated gene during development of the chicken oviduct and that it may be a candidate gene for further research on its role in tumorigenesis leading to EOC in the laying hen.

## Results

### Multiple sequence alignment, pairwise comparisons, and phylogenetic analysis

**Figure 1:**
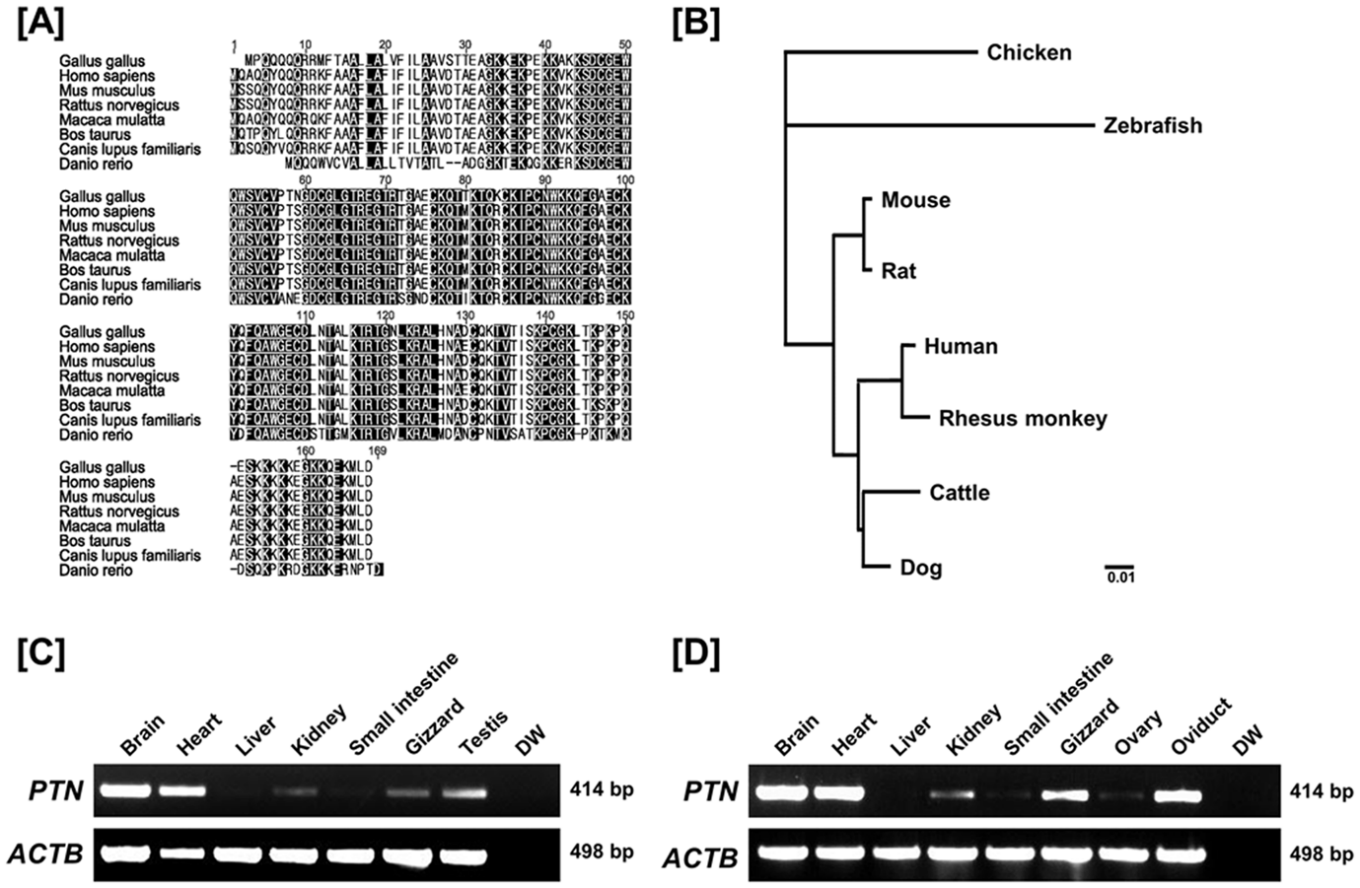
Multiple sequence alignment and tissue-specific expression of *PTN* in chickens. [A] The amino acid sequences of PTN from each of seven species (chicken, human, rhesus monkey, mouse, rat, cattle, dog and zebrafish) are presented based on alignments determined using Geneious Alignment [76] with BLOSUM (Blocks Substitution Matrix) and gap penalties. Amino acid sequences in the shaded boxes represent those that are identical among sequences for chicken and mammalian PTN and dashes indicate gaps in the sequences. [B] The phylogenetic tree of PTN generated from alignments of primary sequences of chicken, human, rhesus monkey, mouse, rat, cattle, dog and zebrafish PTN proteins using bootstrap analysis with 1,000 replicates. [C and D] Expression of PTN in various organs of male and female of chickens. Results of RT-PCR analysis using cDNA templates from different organs of male [C] and female [D] chickens with chicken *PTN* and chicken *ACTB*-specific primers. *See* *Materials and Methods* *for complete description*.

The *PTN* gene was found to span 66,234 bp on chicken chromosome 1 and consists of three exons. *PTN* mRNA has 2,551 bp encoding a protein with 165 amino acid residues (Figure 1A). The amino acid sequence of chicken *PTN* was compared to those of eight other species. The pair-wise comparisons of *PTN* orthologs revealed that chicken PTN protein is well conserved with high homology relative to other mammalian PTN proteins (90.9 to 92.6%, Table 1). The phylogenetic tree constructed with the neighbor-joining method is presented in Figure 1B. The human and rhesus monkey *PTN* genes clustered together and formed a larger cluster with cattle and dog, and an even larger cluster with sister groups was detected for mouse and rat. However, chicken PTN is in a separate branch, but closer to zebrafish than to other species. These results indicate that chicken PTN diverged from mammalian PTNs at very early stage in its evolution.

**Table 1:** Pairwise comparison of PTN between chicken and other species.

Species	Symbol	Identity (%)
Chicken (*Gallus gallus*)	PTN	-
vs. Human (*Homo sapiens*)	PTN	91.4
vs. Rhesus monkey (Macaca mulatta)	PTN	91.0
vs. Mouse (*Mus musculus*)	Ptn	92.6
vs. Rat (*Rattus norvegicus*)	Ptn	92.6
vs. Cattle (*Bos taurus*)	PTN	90.9
vs. Dog (*Canis lupus familiaris*)	PTN	91.4
vs. Zebrafish (*Danio rerio*)	ptn	65.4

### Expression pattern of PTN mRNA in various organs from chickens

Tissue specific expression of *PTN* mRNA in brain, heart, liver, kidney, small intestine, gizzard, ovary, oviduct and testis of 1- to 2-year-old male (n = 3) and female (n = 3) chickens was determined by RT-PCR analyses. Results indicated high levels of expression of *PTN* mRNA in brain and heart from male and brain, heart, gizzard and oviduct from female chickens (Figures 1C and 1D), and lower expression in kidney, gizzard and testis from males and kidney and ovary from females. However, expression of *PTN* mRNA was not detected in other organs analyzed. We reported differential gene profiling of the chicken oviduct [16] and found that the avian homolog of the human *PTN* transcript is highly expressed in chicks treated with diethylstilbestrol (DES, a synthetic estrogen agonist). However, little is known about expression and function of PTN in the oviduct of any species, so this study focused on the chicken oviduct.

### Localization of chicken PTN mRNA and protein in chicken oviduct

**Figure 2:**
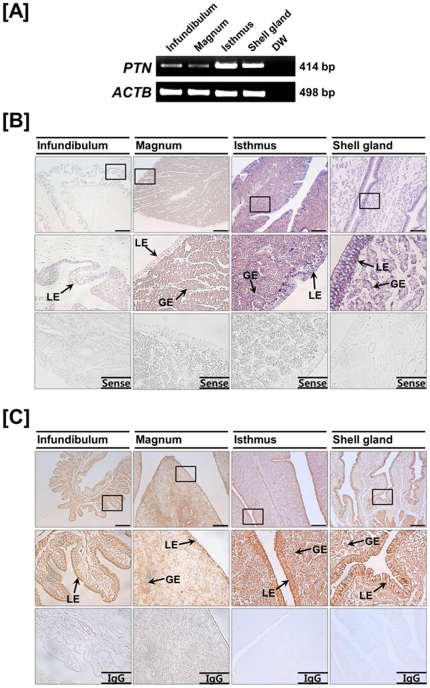
Expression of PTN in the chicken oviduct. [A] Results of RT-PCR analysis using cDNA templates from each segment of the chicken oviduct with chicken *PTN* and chicken *ACTB*-specific primers. [B] *In situ* hybridization analyses of *PTN* mRNAs in the chicken oviduct. Cross-sections of the infundibulum, magnum, isthmus and shell gland of the chicken oviduct were hybridized with antisense or sense chicken *PTN* cRNA probes. [C] Immunoreactive PTN protein in the chicken oviduct. For the IgG control, normal rabbit IgG was substituted for the primary antibody. Sections were not counterstained. Legend: LE, luminal epithelium; GE, glandular epithelium. *Scale bar* represents 100 µm. *See* *Materials and Methods* *for complete description*.

Structurally, the oviduct of egg-laying hens includes the infundibulum (site of fertilization), magnum (production of components of egg-white), isthmus (formation of the shell membrane), and shell gland (formation of the egg shell). Results of RT-PCR analysis showed abundant level of *PTN* mRNA in isthmus and shell gland compared to infundibulum and magnum (Figure 2A). To determine cell-specific localization of *PTN* mRNA in the chicken oviduct, *in situ* hybridization analysis was performed. As illustrated in Figure 2B, *PTN* mRNA was most abundant in the glandular (GE) and luminal (LE) epithelia of the isthmus and shell gland, and it was expressed at lower abundance in GE of the magnum. Little or no mRNA was detected in stromal cells, blood vessels, immune cells or myometrium of the oviduct. In addition, results of immunohistochemical analysis (Figure 2C) were consistent with results from *in situ* hybridization analyses in that PTN protein was abundant in LE of the isthmus and shell gland and also detected, to a lesser extent, in the infundibulum and magnum. The nonspecific rabbit IgG, used as a negative control, did not detect any PTN protein.

### Effects of DES on PTN mRNA and protein expression in the chicken oviduct

**Figure 3:**
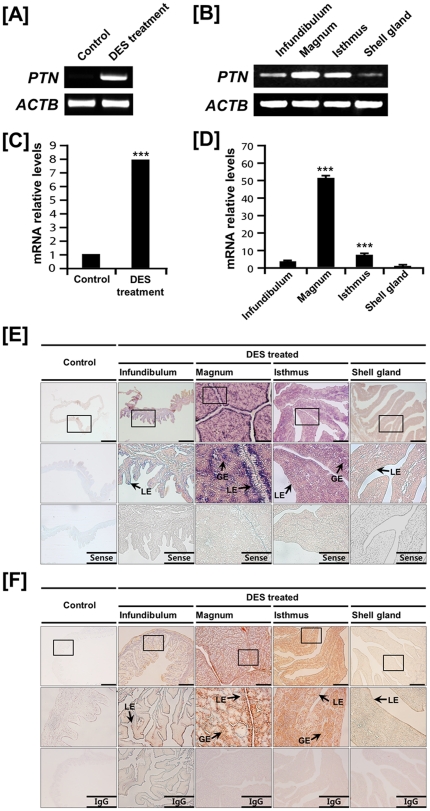
Effect of DES on tissue- and cell-specific expression of PTN in the chicken oviduct. Both RT-PCR [A and B] and quantitative-PCR [C and D] analyses were performed using cDNA templates from DES-treated and control chicken oviducts (mean ± SEM; P<0.001). These experiments were was conducted in triplicate and normalized to control *GAPDH* expression. [E] *In situ* hybridization analyses indicate cell-specific expression of *PTN* mRNA in oviducts of DES-treated and control chicks. Cross-sections of the infundibulum, magnum, isthmus, and shell gland of oviducts from chicks treated with DES or vehicle were hybridized with antisense or sense chicken *PTN* cRNA probes. [F] Immunoreactive PTN protein in oviducts of DES-treated and control chicks. For the IgG control, normal rabbit IgG was substituted for the primary antibody. Sections were not counterstained. Legend: LE, luminal epithelium; GE, glandular epithelium. *Scale bar* represents 100 µm. *See* *Materials and Methods* *for complete description*.

Cell-type specific expression of PTN in the oviductal segments of mature hens suggested regulation by estrogen during development of the chick oviduct. We reported that administration of exogenous DES stimulates growth, development and cytodifferentiation of the postnatal chick oviduct and found several candidate genes and pathways related to the regulation of oviduct development [16]. Therefore, we examined effects of DES on PTN expression in the chick oviduct. As illustrated in Figures 3A and 3B, semi-quantitative RT-PCR analysis indicated that DES treatment increased *PTN* mRNA levels in all segments of the chick oviduct. Further results from quantitative PCR revealed that DES induced an 8.1-fold increase (P<0.001) in oviductal *PTN* mRNA as compared to control chicks (Figure 3C). In addition, DES stimulated 3.6-, 51.1-, and 6.9-fold increases (P<0.001) in *PTN* mRNA in the infundibulum, magnum and isthmus, respectively (Figure 3D). As shown in Figure 3E, *in situ* hybridization analyses revealed that *PTN* mRNA is expressed specifically in GE and LE of the magnum and isthmus of chick oviducts treated with DES and, at lower abundance in the shell gland and infundibulum. Consistent with these results, immunoreactive PTN protein was detected predominantly in GE and LE of magnum and isthmus, and to a lesser extent, in infundibulum and shell gland (Figure 3F).

### Post-transcriptional regulation of microRNAs affecting PTN

**Figure 4:**
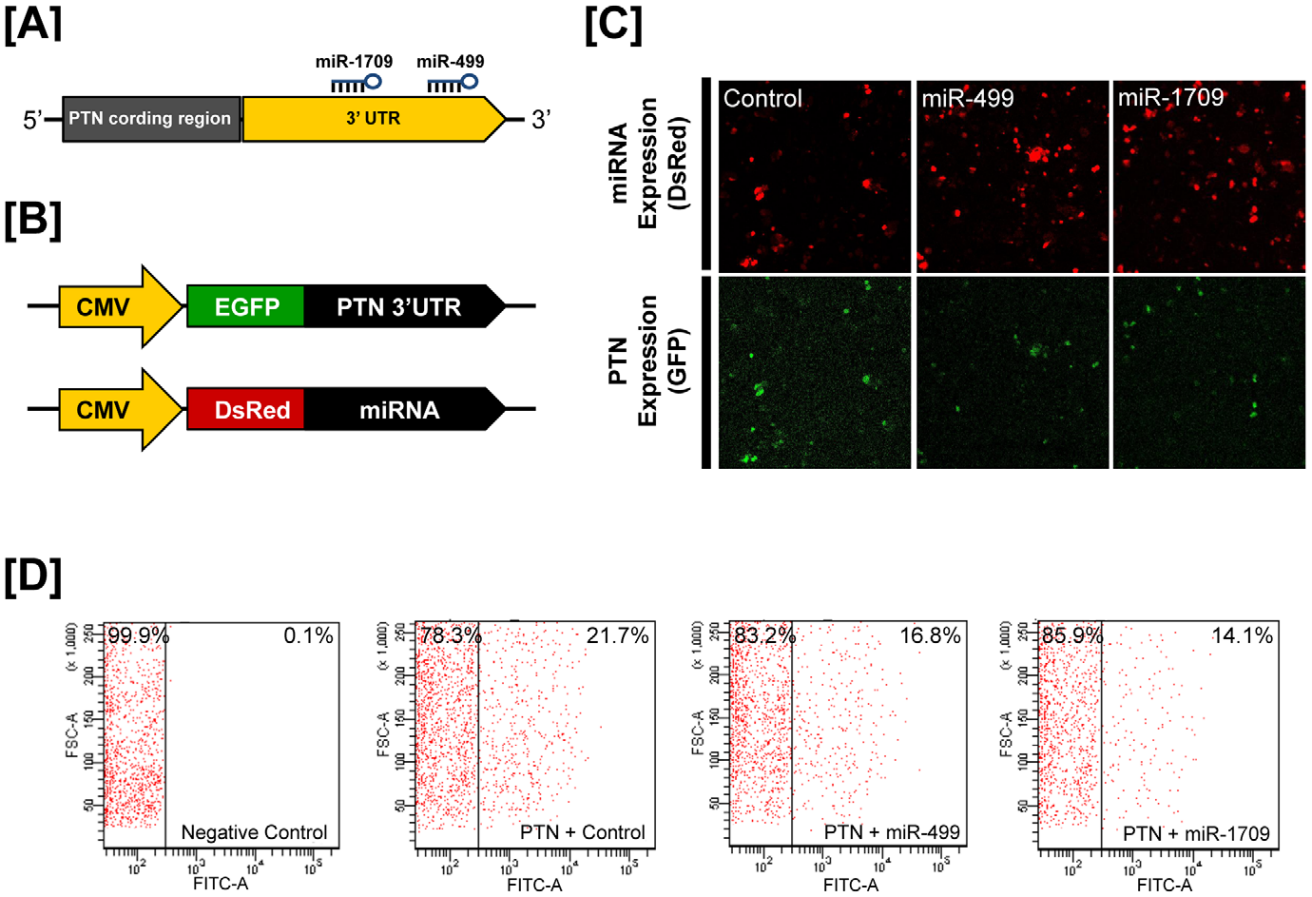
*In vitro* target assay of *microRNAs* on the *PTN* transcript. [A] Diagram of *miR-499* and *miR-1709* binding sites in *PTN* 3′-UTR. [B] Expression vector maps for eGFP with *PTN* 3′-UTR and Ds-Red with each miRNA. The 3′-UTR of the *PTN* transcript was subcloned between the *eGFP* gene and the polyA tail to generate the fusion construct of the GFP transcript following the miRNA target 3′-UTR (pcDNA-eGFP-3′UTR) (upper panel) and the miRNA expression vector was designed to co-express DsRed and each miRNA (pcDNA-DsRed-miRNA) (lower panel). [C and D] After co-transfection of pcDNA-eGFP-3′UTR for the *PTN* transcript and pcDNA-DsRed-miRNA for the *miR-499* and *miR-1709*, the fluorescence signals of GFP and DsRed were detected using FACS [C] and fluorescent microscopy [D]. See *Materials and Methods* for complete description.

To investigate the possibility that *PTN* expression is regulated at the post-transcriptional level by miRNAs, we performed a miRNA target validation assay. Analysis of potential miRNA binding sites within the 3′-UTR for *PTN* using a miRNA target prediction database (miRDB; http://mirdb.org/miRDB/) revealed six putative binding site for *miR-499*, *miR-1555*, *miR-1632*, *miR-1709*, *miR-1787* and *miR-1815*. Therefore, we determined if these six miRNAs influenced PTN expression via its 3′-UTR. A fragment of the PTN 3′-UTR harboring binding sites for the miRNAs were cloned downstream of the green fluorescent protein (GFP) reading frame, thereby creating a fluorescent reporter for function of the 3′-UTR region. After co-transfection of eGFP-*PTN* 3′-UTR and DsRed-miRNA, the intensity of GFP expression and percentage of GFP-expressing cells were analyzed by fluorescence microscopy and FACS. As shown in Figures 4C and 4D, in the presence of *miR-499* and *miR-1709*, the intensity and percentage of GFP-expressing cells (21.7% in control vs. 14.1% in *miR-499*, 16.8% in *miR-1709*) decreased (p<0.01). On the other hand, in the presence of *miR-1555*, *miR-1632*, *miR-1787* and *miR-1815*, there was no significant decrease in green fluorescence as compared to the control (data not shown). These results indicate that at least two miRNAs directly bind to the *PTN* transcript and post-transcriptionally regulate *PTN* gene expression.

### Differential expression of PTN in normal and cancerous ovaries of hens

**Figure 5:**
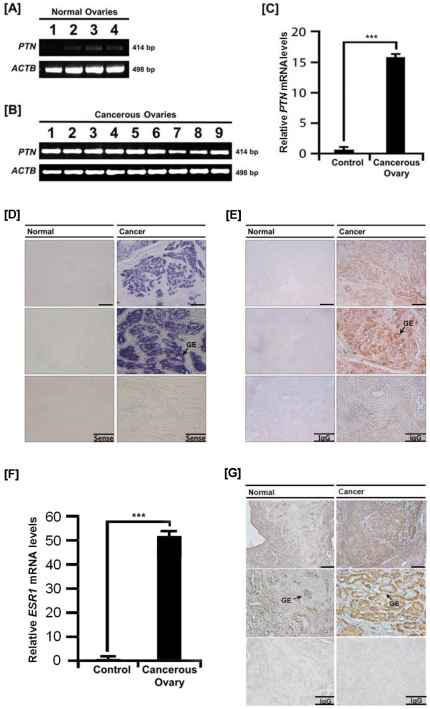
Quantitation of expression of PTN and ESR1 in normal and cancerous ovaries from hens. [A] RT-PCR analyses were performed using cDNA templates from normal and cancerous ovaries of laying hens using chicken *PTN* and *ACTB*-specific primers. Lanes 1 to 4 show results of analyses of four normal ovaries. [B] Lanes 1–9 are from analyses of 9 different cancerous ovaries from laying hens. Expression of *PTN* mRNA was abundant in all carcinomas, but not in normal ovaries. Legend for panel B: Lane 1, endometrioid/serous/mucinous carcinoma (Stage III); Lane 2, endometrioid carcinoma (Stage I); Lane 3, serous carcinoma (Stage I); Lane 4, mucinous/endometrioid carcinoma (Stage IV); Lane 5, endometrioid carcinoma (Stage IV); Lane 6, endometrioid carcinoma (Stage III); Lane 7, clear cell carcinoma (Stage IV); Lane 8, serous/mucinous carcinoma (Stage IV); and Lane 9, serous/mucinous/endometrioid carcinoma (Stage III) [21]. [C] The q-PCR analysis for *PTN* mRNA was performed using cDNA templates from normal and cancerous ovaries of laying hens (mean ± SEM; P<0.001). [D] *In situ* hybridization analyses of *PTN* mRNA in normal and cancerous ovaries of hens. Cross-sections of normal and cancerous ovaries of hens hybridized with antisense or sense chicken *PTN* cRNA probes demonstrated abundant *PTN* mRNA predominantly in GE of cancerous ovaries, but not in LE, stroma or blood vessels. [E] Immunoreactive PTN protein in normal and cancerous ovaries of hens. For the IgG control, normal rabbit IgG was substituted for the primary antibody. Sections were not counterstained. Legend: GE, glandular epithelium. *Scale bar* represents 200 µm (the first horizontal panels, sense and IgG) or 50 µm (the second horizontal panels, sense and IgG). [F] The q-PCR analysis for expression of *estrogen receptor alpha (ESR1)* was performed using cDNA templates from normal and cancerous ovaries of laying hens (mean ± SEM; P<0.001). [G] Immunoreactive ESR1 protein in normal and cancerous ovaries of hens. For the IgG control, normal rabbit IgG was substituted for the primary antibody. Sections were not counterstained. Legend: GE, glandular epithelium. *Scale bar* represents 200 µm (the first horizontal panels, sense and IgG) or 50 µm (the second horizontal panels, sense and IgG). *See* *Materials and Methods* *for a complete description of the methods*.

Chickens are considered the most relevant animal model to identify and develop biomarkers for patients with epithelial ovarian cancer because their incessant ovulation increases the possibility of gene mutations by genomic damage to the ovarian surface epithelium which can lead to ovarian cancer [30]. We previously reported expression of cysteine protease cathepin B (CTSB), serpin peptidase inhibitor, clade B, member 11 (SERPINB11) and alpha 2 macroglobulin (A2M) in ovarian tissue from hens with ovarian cancer [20], [21], [36]. Based on similarities among these genes in expression patterns and estrogen-mediated regulation in the oviduct, we hypothesized that expression patterns for PTN would differ between normal and cancerous ovarian tissues from hens. Based on RT-PCR analysis, *PTN* mRNA was found in all carcinomas, but there was little or no expression in normal ovaries (Figure 5A and 5B). Further, the level of expression of PTN mRNA was greater (P<0.001) in cancerous ovaries from hens (Figure 5C).

### Localization of PTN mRNA and protein in cancerous ovaries of hens

To determine cell-specific expression of PTN mRNA and protein, *in situ* hybridization analysis and immunohistochemistry were performed. As illustrated in Figure 5D, there was abundant *PTN* mRNA localized predominantly in GE of cancerous ovaries, but not in LE, stroma or blood vessels. Consistent with this result, immunoreactive PTN protein was detected in GE of cancerous ovaries, but not in any other cell types of the ovaries (Figure 5E). Furthermore, we determined if the increased expression of PTN in cancerous tissue was correlated with changes in circulating concentrations of estradiol in plasma and expression of estrogen receptor alpha (*ESR1*) in normal and cancerous ovaries. Even though in our preliminary experiment we could not detect any differences in concentrations of estradiol in serum between cancerous and non-cancerous groups (0.18±0.1 ng/ml vs. 0.17±0.1 ng/ml), quantitative PCR analysis revealed that *ESR1* mRNA levels increased 51-fold (*P*<0.001) in cancerous ovaries, but not in normal ovaries (Figure 5F). In addition, immunohistochemical analysis showed that immunoreactive ESR1 was abundant in the GE of cancerous ovaries (Figure 5G). Little or no ESR1 was detected in normal ovaries. These results indicate that PTN abundance increases in response to estrogen in cancerous ovaries of hens.

### Comparison of CpG methylation status in the upstream of PTN gene between normal and cancerous ovarian cells in hens

**Figure 6:**
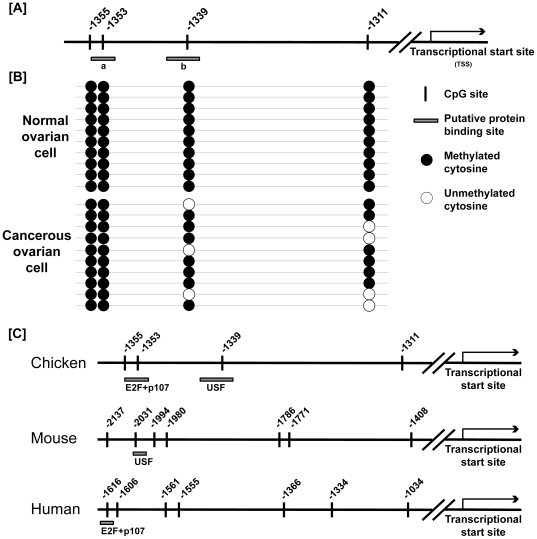
Bisulfite sequencing of CpG sites in the upstream region of the *PTN* gene. [A] Schematic of the four CpG sites in the promoter region of the PTN gene are indicated by the heavy black vertical lines. The numbers on the line indicate positions relative to the transcription start site. Legend: a, complex contains E2F and p107 (E2F+p107); b, upstream stimulatory factor (USF also known as gamma-factor). [B] The CpG methylation status in the upstream region of the *PTN* gene was analyzed in normal and ovarian cancer cells from hens by bisulfate sequencing. Each circle indicates a CpG site in the primary sequence, and each line of circles represents analysis of a single cloned allele. Closed and open circles are methylated and unmethylated CpGs, respectively. [C] Comparison of the sequences around CpG regions of *PTN* genes of chicken, mouse, and human. The 5′ flanking region (about 2.2 kb) of mouse and human *PTN* was compared to that of chicken *PTN* to identify differences in sequences around each CpG sites among those species.

The CpG methylation status in the upstream region of genes regulates its transcriptional activity and it is closely associated with the initiation and growth of carcinomas. Therefore, we investigated methylation patterns in the promoter region of the *PTN* gene in normal and cancerous ovarian epithelial cells. Both normal and cancerous epithelial cells were extracted and cultured *in vitro* as previously reported [37]. Results of bisulfite sequencing indicated that CpG sites at −1,353 and −1,355 CpG from the transcriptional start site remained methylated in both normal and cancerous cells. However, 30- and 40% of −1,339 and −1,311 CpG sites were demethylated in ovarian cancer cells, but not in normal ovarian epithelial cells (Figure 6A and 6B).

## Discussion

Results of the current study are novel in providing the first comparisons among chicken and mammalian *PTN* genes with respect to structure, phylogenetic evolution, tissue specific expression of PTN mRNA and protein, and regulation of expression by estrogen in the chicken oviduct. Our results also revealed that *PTN* gene expression is post-transcriptionally regulated by several miRNAs critical to development of the chick oviduct in response to estrogen. These results support our hypothesis that PTN is required for growth, development and functional aspects of the mature oviduct of hens in response to estrogen during their reproductive cycle. Our previous report on differential gene profiling of the chick oviduct indicated that the avian homolog of the human *PTN* transcript is highly expressed in chicks treated with DES [34].

The PTN and midkine (MK) proteins are members of a family of proteins regulating growth and differentiation which share over 50% amino acid sequence identity and their genes are located on human chromosomes 7q.33 and 11p.11.2, respectively [3], [38]. In chickens, MK (also called retinoic acid-inducible heparin binding protein) purified from 11-day-old chicken embryos is predominantly localized within the basement membranes in embryonic tissues and it stimulates neurite outgrowth and proliferation of PC12 cells [39], [40]. However, little is known about the expression and function of PTN in chickens. In the present study, multiple gene sequence alignment showed that the *PTN* gene in the chicken genome spans 66,234 bp on chromosome 1 and consists of 3 exons (2,551 bp mRNA) encoding a protein with 165 amino acid residues (Figure 1A). The human *PTN* gene is also about 68 kb [41], although the human *MK* genes is only 2 kb [42]. In addition, results of the present study indicated that chicken PTN protein is highly conserved with respect to mammalian PTN proteins (90.9 to 92.6%) and that it diverged from mammalian PTN very early in its evolution.

As illustrated in Figure 1D, *PTN* mRNA is abundantly expressed in the chicken oviduct. In mice, the *Ptn* gene is detected in a number of tissues and co-localizes with the *Mk* gene in many cases during embryogenesis [43]. Although PTN has important roles in various biological events such as differentiation of renal tubular epithelial cells and dopaminergic neurons [44], [45], *Ptn^−/−^* mice are fertile and exhibit no gross anatomical abnormalities except for abnormal structure and function of components of the nervous system [46], [47]. Recently, Muramatsu and collegues [56] reported that mice deficient in both *Ptn* and *Mk* were infertile and had abnormal estrous cycles with long periods of proestrus and diestrus and short periods of estrus [48]. In addition, the presence of PTN in the porcine uterus and uterine flushings during early pregnancy may be a member of the regulators of implantation and conceptus development [49], [50]. In the present study, PTN mRNA and protein were most abundant in the LE of the isthmus and shell gland, but detectable at lower abundance in GE in each segment. As shown in Figure 3, q-PCR analyses revealed that DES induced oviductal *PTN* mRNA to a 51.1-fold increase as compared to control chicks (P<0.001) in the magnum. In chickens, estrogen stimulates proliferation and cytodifferentiation of immature oviductal epithelial cells to mature tubular gland cells to activate egg white protein genes during oviduct development [12], [13]. Of particular note, the fully differentiated tubular gland cells of the magnum produce and secrete several egg-white proteins such as ovalbumin, ovomucoid, ovotransferrin and avidin during egg formation and oviposition [51]. Therefore, the magnum is the most estrogen-responsive portion of the chicken oviduct that affects the quality of the egg. These results suggest that the functional role(s) of PTN in the LE of the magnum is similar with those in the porcine uterus during early pregnancy.

In a wide variety of fundamental processes and biological events in vertebrates, such as cellular survival, growth, development and differentiation, microRNAs (miRNAs) play pivotal roles in post-transcriptional regulation and pathways [52]. As shown in Figure 4, co-transfection of eGFP-*PTN* 3′-UTR and DsRed-miRNA decreased the percentage of GFP-positive cells and GFP fluorescence density in *miR-499* and *miR-1709* transfected cells, but not in cell transfected with *miR-1555*, *miR-1632*, *miR-1787* and *miR-1815* when compared to controls. These results indicate that *miR-499* and *miR-1709* bind directly to the 3′-UTR of the *PTN* transcript and post-transcriptionally regulate *PTN* gene transcription. Thus, we propose that these two miRNAs are closely related to the regulatory pathways of oviduct development and differentiation in chickens; however, this requires further investigation.

Results of the present study are the first to identify a high level of expression of the *PTN* gene in GE of ovarian carcinoma in laying hens. Indeed, cancerous ovaries of hens show very similar patterns of expression of tumor-related genes compared with those in women [53], and high cross-reactivity and expression of biomarkers such as CA125, EGFR, and ERBB-2 for human ovarian cancer [54], [55], [56], [57]. Therefore, laying hens are the most relevant animal model to identify biomarkers for patients with epithelial ovarian cancer. Indeed, we found that cathepin B (CTSB) [20], serpin peptidase inhibitor, clade B, member 11 (SERPINB11) [21] and alpha 2 macroglobulin (A2M) [36] genes are most abundant in GE of chicken adenocarcinoma. Likewise, we now report that the *PTN* gene is expressed predominantly in GE of the cancerous ovaries from hens. Moreover, we recently reported that SERPINB3 is a biomarker for chicken ovarian endometrioid carcinoma and that it can serve as a prognostic factor for platinum resistance and poor survival in patients with epithelial ovarian cancer [58]. On the other hand, results of the the present study revealed differences in the methylation status of CpG sites in the promoter region of the *PTN* gene in surface epithelial cells of cancerous ovaries. In general, a number of genes are up- and down-regulated in cancer cells of various origins via DNA methylation and histone modification [59]. These epigenetic regulatory mechanisms stimulate rates of tumor growthand metastasis by activation of oncogenes and inactivation of tumor suppressor genes through differential methylation of genes in the promoter region [60], [61]. In the present study, results of bisulfite sequencing revealed that 30- and 40% of −1,339 and −1,311 CpG sites were demethylated in ovarian cancer cells, but not in normal ovarian epithelial cells (Figure 6). This different metylation status from between normal and cancerous ovarian cells likely affects development of cancer phenotypes. In addition, expression of the *PTN* gene may be epigenetically regulated, and its cell type specific expression closely associated with DNA methylation. As illustrated in Figure 6C, the position of the −1,339 CpG site is located within the putative binding elements for the upstream stimulatory factor (USF also known as gamma-factor). Furthermore, we compared the 5′ flanking region (about 2.2 kb) of mouse and human PTN with that of chicken PTN to identify differences in sequences around CpG sites and found one CpG site in mouse, but not in human. However, further research is required to elucidate the relationship between PTN and USF signaling cascades in cancerous ovaries of hens. These results support our hypothesis that PTN is a critical regulator for growth and developmental aspects of epithelial cells of the ovaries of laying hens as they transition from a normal to a cancerous state. In humans, the *PTN* gene is involved in carcinogenesis including mitogenesis, metastasis and angiogenesis, and it is expressed in a variety of cancers, such as lung [10], [62], colon [63], prostate [8], breast [7] and pancreas [64] as well as melanomas [9], neuroblastomas [65] and many carcinoma cell lines. It is thought that PTN is an angiogenic factor thatstimulates tumor growth and metastasis. For instance, nude mice implanted with breast cancer cells and treated with dominant negative PTN showed a significant decrease in the rates of tumor growth and angiogenesis [66], whereas mice overexpressing PTN in breast cancer cells showed increased levels of tumor growth, microvessel density and endothelial cell proliferation [67]. These results indicate that PTN secreted from tumor cells stimulate proliferation and tube formation of endothelial cells [4].

Collectively, results of the present study indicate that *PTN* is a novel estrogen-stimulated gene during growth, development and differentiation of the chicken oviduct and that it is likely a critical regulator of abnormal growth and functional aspects of ovarian surface epithelial cells as they transition from normal to a cancerous state in laying hens. These results also provide important insight into future research to investigate the precise role(s) and signal transduction cascades involving PTN. Research will be directed toward understanding mechanisms responsible for estrogen-mediated development and cytodifferentiation of cells of the chicken oviduct and the significance of PTN as a biomarker of epithelial ovarian cancer of laying hens to elucidate the etiologies and pathogenesis of the disease.

## Materials and Methods

### Experimental Animals and Animal Care

The experimental use of chickens for this study was approved by the Institute of Laboratory Animal Resources, Seoul National University (SNU-070823-5). White Leghorn (WL) laying hens and chicks were subjected to standard management practices at the University Animal Farm, Seoul National University, Korea. The management, reproduction, and embryo manipulation procedures adhered to standard operating protocols of our laboratory. All chickens were exposed to a light regimen of 15 h light and 9 h dark with *ad libitum* access to feed and water.

### Tissue Samples

#### Study one

Following euthanasia of mature WL hens, tissue samples were collected from brain, heart, liver, kidney, small intestine, gizzard, ovary, oviduct and testis of 1- to 2- year-old males (n = 3) and females (n = 3). Subsets of these samples were frozen or fixed in 4% paraformaldehyde for further analyses. Frozen tissue samples were cut into 5- to 7-mm pieces, frozen in liquid nitrogen vapor, and stored at −80°C. The other samples were cut into 10 mm pieces and fixed in fresh 4% paraformaldehyde in PBS (pH 7.4). After 24 h, fixed tissues were changed to 70% ethanol for 24 h and then dehydrated and embedded in Paraplast-Plus (Leica Microsystems, Wetzlar, Germany). Paraffin-embedded tissues were sectioned at 5 µm.

#### Study two

Female chicks were identified by PCR analysis using W chromosome-specific primer sets [68]. Treatment with DES and recovery of the oviduct were conducted as reported previously [69], [70]. Briefly, a 15 mg DES pellet was implanted subcutaneously in the abdominal region of 1-week-old female chicks for release of hormone for 20 days [69], [71], [72]. Five chicks in each group were euthanized using 60%–70% carbon dioxide. Subsets of these samples were frozen or fixed in 4% paraformaldehyde for further analyses. Frozen tissue samples were cut into 5- to 7-mm pieces and frozen in liquid nitrogen. The other samples were cut into 10- to 15-mm pieces and fixed in fresh 4% paraformaldehyde in PBS (pH 7.4). After 24 h, fixed tissues were changed to 70% ethanol for 24 h and then dehydrated and embedded in Paraplast-Plus (Leica Microsystems, Wetzlar, Germany). Paraffin-embedded tissues were sectioned at 5 µm.

#### Study three

In this study, a total of 136 chickens (88 chickens over 36 months of age and 48 chickens over 24 months of age), which had completely stopped laying eggs were euthanized for biopsy and cancerous (n = 10) ovaries were collected. As a control, normal (n = 5) ovaries were also collected from egg-laying hens. We examined tumor stage in 10 hens with cancerous ovaries based on characteristic features of chicken ovarian cancers [17]. In three hens, ovarian tumor cells were classified as Stage III as they had metastasized to the gastrointestinal tract and superficial surface of the liver with profuse ascites in the abdominal cavity. In five hens, the tumors had metastasized to distant organs such as liver parenchyma, lung, gastrointestinal tract and oviduct with profuse ascites, so these were classified at Stage IV tumors. The other two hens did not have tumors in any other organs; therefore, their ovarian tumors were classified as Stage I. Subsets of these samples were frozen or fixed in 4% paraformaldehyde for further analyses. Frozen tissue samples were cut into 5- to 7-mm pieces and frozen in liquid nitrogen. The other samples were cut into 10 mm pieces and fixed in 4% paraformaldehyde in PBS (pH 7.4). After 24 h, fixed tissues were changed to 70% ethanol for 24 h and then dehydrated and embedded in Paraplast-Plus (Leica Microsystems, Wetzlar, Germany). Paraffin-embedded tissues were sectioned at 5 µm and stained with hematoxylin and eosin. Epithelial ovarian cancers in chickens were classified based on their cellular subtypes and patterns of cellular differentiation with reference to ovarian malignant tumor types in humans [17].

### RNA Isolation

Total cellular RNA was isolated from frozen tissues using Trizol reagent (Invitrogen, Carlsbad, CA) according to manufacturer's recommendations. The quantity and quality of total RNA was determined by spectrometry and denaturing agarose gel electrophoresis, respectively.

### Semiquantitative RT-PCR analysis

The expression of *PTN* mRNA in normal and cancerous ovaries of chickens, was assessed using semi-quantitative RT-PCR as described previously [73]. The cDNA was synthesized from total cellular RNA (2 ug) using random hexamer (Invitrogen, Carlsbad, CA) and oligo (dT) primers and AccuPower® RT PreMix (Bioneer, Daejeon, Korea). The cDNA was diluted (1∶10) in sterile water before use in PCR. For *PTN*, the sense primer (5′-TGC TCT CCT GGC ACT TGT CT-3′) and antisense primer (5′-CTT GGA TTC TTG AGG TTT GGG-3′) amplified a 414-bp product. For *ACTB* (housekeeping gene; *beta-actin*), the sense primer (5′-GTG TGA TGG TTG GTA TGG GC-3′) and antisense primer primer (5′-TTT CTC TCT CGG CTG TGG TG-3′) amplified a 498-bp product. The primers, PCR amplification and verification of their sequences were conducted as described previously [73]. PCR amplification was conducted using approximately 60 ng cDNA as follows: (1) 95°C for 3 min; (2) 95°C for 20 sec, 60°C for 40 sec (for *PTN* and *ACTB*) and 72°C for 1 min for 35 cycles; and (3) 72°C for 10 min. After PCR, equal amounts of reaction product were analyzed using a 1% agarose gel, and PCR products were visualized using ethidium bromide staining. The amount of DNA present was quantified by measuring the intensity of light emitted from correctly sized bands under ultraviolet light using a Gel Doc™ XR+ system with Image Lab™ software (Bio-Rad).

### Quantitative RT-PCR Analysis

Total RNA was extracted from each sample of normal and cancerous ovarian tissue from hens using TRIzol (Invitrogen) and purified using an RNeasy Mini Kit (Qiagen). Complementary DNA was synthesized using AccuPower® RT PreMix (Bioneer, Daejeon, Korea). Gene expression levels were measured using SYBR® Green (Sigma, St. Louis, MO, USA) and a StepOnePlus™ Real-Time PCR System (Applied Biosystems, Foster City, CA, USA). The *GAPDH* gene was simultaneously analyzed as a control and used for normalization for variation in loading. Each target gene and *GAPDH* was analyzed in triplicate. Using the standard curve method, we determined the level of expression of the examined genes using the standard curves and C_T_ values, and normalized them using *GAPDH* expression quantities. The PCR conditions were 95°C for 3 min, followed by 40 cycles at 95°C for 30 sec, 60°C for 30 sec, and 72°C for 30 sec using a melting curve program (increasing the temperature from 55°C to 95°C at a rate of 0.5°C per 10 sec) and continuous fluorescence measurement. ROX dye (Invitrogen) was used as a negative control for the fluorescence measurements. Sequence-specific products were identified by generating a melting curve in which the C_T_ value represented the cycle number at which a fluorescent signal was statistically greater than background, and relative gene expression was quantified using the 2^−ΔΔCT^ method [74]. For the control, the relative quantification of gene expression was normalized to the C_T_ of the control oviduct.

### *In Situ* Hybridization Analysis

For hybridization probes, PCR products were generated from cDNA with the primers used for RT-PCR analysis. The products were gel-extracted and cloned into TOPO® vector (invitrogen). After verification of the sequences, plasmids containing the correct gene sequences were amplified with T7- and SP6-specific primers (T7:5′-TGT AAT ACG ACT CAC TAT AGG G-3′; SP6:5′-CTA TTT AGG TGA CAC TAT AGA AT-3′) then digoxigenin (DIG)-labeled RNA probes were transcribed using a DIG RNA labeling kit (Roche Applied Science, Indianapolis, IN). Tissues were collected and fixed in 4% paraformaldehyde, embedded in paraffin and sectioned at 5 µm on APES-treated (silanized) slides. The sections were then deparaffinized in xylene and rehydrated to diethylpyrocarbonate (DEPC)-treated water through a graded series of alcohol. The sections were treated with 1% Triton X-100 in PBS for 20 min and washed two times in DEPC-treated PBS. After washing in DEPC-treated PBS, they were digested with 5 µg/ml proteinase K (Sigma) in TE buffer (100 mM Tris-HCl, 50 mM EDTA, pH 8.0) at 37°C. After postfixation in 4% paraformaldehyde, sections were incubated twice for 5 min each in DEPC-treated PBS and incubated in TEA buffer (0.1M triethanolamine) containing 0.25% (v/v) acetic anhydride. The sections were incubated in a prehybridization mixture containing 50% formamide and 4× standard saline citrate (SSC) for at least 10 min at room temperature. After prehybridization, the sections were incubated with a hybridization mixture containing 40% formamide, 4× SSC, 10% dextran sulfate sodium salt, 10 mM DTT, 1 mg/ml yeast tRNA, 1 mg/ml salmon sperm DNA, 0.02% Ficoll, 0.02% polyvinylpyrrolidone, 0.2 mg/ml RNase-free bovine serum albumin and denatured DIG-labeled cRNA probe for overnight at 42°C in a humidified chamber. After hybridization, sections were washed for 15 min in 2× SSC at 37°C, 15 min in 1× SSC at 37°C, 30 min in NTE buffer (10 mM Tris, 500 mM NaCl and 1 mM EDTA) at 37°C and 30 min in 0.1× SSC at 37°C. After blocking with a 2% normal sheep serum (Santa Cruz Biotechnology, INC.), the sections were incubated overnight with sheep anti-DIG antibody conjugated to alkaline phosphatase (Roche). The signal was visualized by exposure to a solution containing 0.4 mM 5-bromo-4-chloro-3-indolyl phosphate, 0.4 mM nitroblue tetrazolium, and 2 mM levamisole (Sigma).

### Immunohistochemistry

Immunocytochemical localization of PTN protein in normal and cancerous ovaries from chickens was performed as described previously [75] using a rabbit polyclonal antibody to PTN (Catalog number ab-95391; AbCam, CA, USA) and a rabbit antiserum to ESR1 (Catalog number E0646; Sigma-Aldrich, St. Louis, MO, USA) at a final dilution of 1∶200 (0.2 µg/ml) and 1∶100 (0.2 µg/ml), respectively. Antigen retrieval was performed using the boiling citrate method for PTN and Pronase E digestion for ESR1 as described previously [75]. Negative controls included substitution of the primary antibody with purified non-immune rabbit IgG at the same final concentration.

### Prediction of Transcription Factor-Binding *cis*-Elements

The presence of transcription factor-binding *cis*-elements within the PTN promoter region was predicted using a bioinformatics tool for orthologous sequences (TFSEARCH ver. 1.3; http://www.cbrc.jp/research/db/TFSEARCH.html).

### Bisulfite Sequencing

DNA samples were prepared using an AccuPrep Genomic DNA Extraction Kit (Bioneer) and converted using Epitect Bisulfite kit (QIAGEN, Doncaster, Australia) according to the manufacturer's instructions. For amplifying the converted DNA, PCRs were performed with forward (5-GGA TTT TTG TGT AAA TTT GGA GTA G-3) and reverse (5-TTC CAA AAT CCA AAC AAT TTC TAT C-3) primers, which included the upstream region of the *PTN* gene, as follows: 95°C for 3 min, 35 cycles at 95°C for 1 min, 56°C for 1 min, 72°C for 2 min, and 72°C for 5 min for the final synthesis. The PCR products were cloned into the pGEM-T easy vector (Promega, Madison, WI) and sequenced using an ABI Prism 3730 XL DNA Analyzer (Applied Biosystems, Foster City, CA).

### MicroRNA Target Validation Assay

The 3′-UTR of *PTN* was cloned and confirmed by sequencing. The 3′-UTR was subcloned between the eGFP gene and the bovine growth hormone (bGH) poly-A tail in pcDNA3eGFP (Clontech, Mountain View, CA) to generate the eGFP-miRNA target 3′-UTR (pcDNA-eGFP-3′UTR) fusion constructs. For the dual fluorescence reporter assay, the fusion constructs containing the *DsRed* gene and either *miR-499*, *miR-1555*, *miR-1632*, *miR-1709*, *miR-1787* or *miR-1815* were designed to be co-expressed under control of the CMV promoter (pcDNA-DsRed-miRNA). The pcDNA-eGFP-3′UTR and pcDNA-DsRed-miRNA (4 µg) were co-transfected into 293FT cells using the calcium phosphate method. When the DsRed-miRNA is expressed and binds to the target site of the 3′-UTR downstream of the GFP transcript, green fluorescence intensity decreases due to degradation of the GFP transcript. At 48 h post-transfection, dual fluorescence was detected by fluorescence microscopy and calculated by FACSCalibur flow cytometry (BD Biosciences). For flow cytometry, the cells were fixed in 4% paraformaldehyde and analyzed using FlowJo software (Tree Star Inc., Ashland, OR).

### Statistical Analyses

Data presented for real-time PCR are expressed as mean ± SEM unless otherwise stated. Differences in the variances between normal and cancerous ovaries were analyzed using the *F* test, and differences between means were subjected to the Student's *t* test. Differences with a probability value of *P*<0.05 were considered statistically significant. Excel (Microsoft, Redmond, WA, USA) was used for statistical analyses.

## References

[1] Milner PG, Li YS, Hoffman RM, Kodner CM, Siegel NR (1989). A novel 17 kD heparin-binding growth factor (HBGF-8) in bovine uterus: purification and N-terminal amino acid sequence.. Biochem Biophys Res Commun.

[2] Li YS, Milner PG, Chauhan AK, Watson MA, Hoffman RM (1990). Cloning and expression of a developmentally regulated protein that induces mitogenic and neurite outgrowth activity.. Science.

[3] Muramatsu T (2002). Midkine and pleiotrophin: two related proteins involved in development, survival, inflammation and tumorigenesis.. J Biochem.

[4] Perez-Pinera P, Chang Y, Deuel TF (2007). Pleiotrophin, a multifunctional tumor promoter through induction of tumor angiogenesis, remodeling of the tumor microenvironment, and activation of stromal fibroblasts.. Cell Cycle.

[5] Meng K, Rodriguez-Pena A, Dimitrov T, Chen W, Yamin M (2000). Pleiotrophin signals increased tyrosine phosphorylation of beta beta-catenin through inactivation of the intrinsic catalytic activity of the receptor-type protein tyrosine phosphatase beta/zeta.. Proc Natl Acad Sci U S A.

[6] Chauhan AK, Li YS, Deuel TF (1993). Pleiotrophin transforms NIH 3T3 cells and induces tumors in nude mice.. Proc Natl Acad Sci U S A.

[7] Fang W, Hartmann N, Chow DT, Riegel AT, Wellstein A (1992). Pleiotrophin stimulates fibroblasts and endothelial and epithelial cells and is expressed in human cancer.. J Biol Chem.

[8] Vacherot F, Caruelle D, Chopin D, Gil-Diez S, Barritault D (1999). Involvement of heparin affin regulatory peptide in human prostate cancer.. Prostate.

[9] Wu H, Barusevicius A, Babb J, Klein-Szanto A, Godwin A (2005). Pleiotrophin expression correlates with melanocytic tumor progression and metastatic potential.. J Cutan Pathol.

[10] Jager R, Noll K, Havemann K, Pfluger KH, Knabbe C (1997). Differential expression and biological activity of the heparin-binding growth-associated molecule (HB-GAM) in lung cancer cell lines.. Int J Cancer.

[11] Dougherty DC, Sanders MM (2005). Estrogen action: revitalization of the chick oviduct model.. Trends Endocrinol Metab.

[12] Socher SH, Omalley BW (1973). Estrogen-Mediated Cell-Proliferation during Chick Oviduct Development and Its Modulation by Progesterone.. Developmental Biology.

[13] Palmiter RD, Wrenn JT (1971). Interaction of Estrogen and Progesterone in Chick Oviduct Development .3.. Tubular Gland Cell Cytodifferentiation. Journal of Cell Biology.

[14] Bar A (2009). Differential Regulation of Calbindin in the Calcium-Transporting Organs of Birds with High Calcium Requirements.. Journal of Poultry Science.

[15] Hincke MT, Nys Y, Gautron J (2010). The Role of Matrix Proteins in Eggshell Formation.. Journal of Poultry Science.

[16] Song G, Seo HW, Choi JW, Rengaraj D, Kim TM (2011). Discovery of candidate genes and pathways regulating oviduct development in chickens.. Biol Reprod.

[17] Barua A, Bitterman P, Abramowicz JS, Dirks AL, Bahr JM (2009). Histopathology of ovarian tumors in laying hens: a preclinical model of human ovarian cancer.. Int J Gynecol Cancer.

[18] Stammer K, Edassery SL, Barua A, Bitterman P, Bahr JM (2008). Selenium-Binding Protein 1 expression in ovaries and ovarian tumors in the laying hen, a spontaneous model of human ovarian cancer.. Gynecol Oncol.

[19] Ansenberger K, Zhuge Y, Lagman JA, Richards C, Barua A (2009). E-cadherin expression in ovarian cancer in the laying hen, Gallus domesticus, compared to human ovarian cancer.. Gynecol Oncol.

[20] Ahn SE, Choi JW, Rengaraj D, Seo HW, Lim W (2010). Increased expression of cysteine cathepsins in ovarian tissue from chickens with ovarian cancer.. Reprod Biol Endocrinol.

[21] Lim W, Kim JH, Ahn SE, Jeong W, Kim J (2011). Avian SERPINB11 Gene: A Marker for Ovarian Cancer in Chickens.. Experimental Biology and Medicine.

[22] Jemal A, Siegel R, Ward E, Murray T, Xu J (2007). Cancer statistics, 2007.. CA Cancer J Clin.

[23] Wong AS, Auersperg N (2003). Ovarian surface epithelium: family history and early events in ovarian cancer.. Reprod Biol Endocrinol.

[24] Cvetkovic D (2003). Early events in ovarian oncogenesis.. Reprod Biol Endocrinol.

[25] Goodman MT, Correa CN, Tung KH, Roffers SD, Cheng Wu X (2003). Stage at diagnosis of ovarian cancer in the United States, 1992–1997.. Cancer.

[26] Pepe MS, Etzioni R, Feng Z, Potter JD, Thompson ML (2001). Phases of biomarker development for early detection of cancer.. J Natl Cancer Inst.

[27] Kurman RJ, Visvanathan K, Roden R, Wu TC, Shih Ie M (2008). Early detection and treatment of ovarian cancer: shifting from early stage to minimal volume of disease based on a new model of carcinogenesis.. Am J Obstet Gynecol.

[28] Kurman RJ, Shih Ie M (2008). Pathogenesis of ovarian cancer: lessons from morphology and molecular biology and their clinical implications.. Int J Gynecol Pathol.

[29] Auersperg N, Wong AS, Choi KC, Kang SK, Leung PC (2001). Ovarian surface epithelium: biology, endocrinology, and pathology.. Endocr Rev.

[30] Murdoch WJ, Van Kirk EA, Alexander BM (2005). DNA damages in ovarian surface epithelial cells of ovulatory hens.. Exp Biol Med (Maywood).

[31] Auersperg N, Edelson MI, Mok SC, Johnson SW, Hamilton TC (1998). The biology of ovarian cancer.. Semin Oncol.

[32] Vanderhyden BC, Shaw TJ, Ethier JF (2003). Animal models of ovarian cancer.. Reprod Biol Endocrinol.

[33] Stakleff KD, Von Gruenigen VE (2003). Rodent models for ovarian cancer research.. Int J Gynecol Cancer.

[34] Song G, Seo HW, Choi JW, Rengaraj D, Kim TM (2011). Discovery of Candidate Genes and Pathways Regulating Oviduct Development in Chickens.. Biol Reprod.

[35] Askew DJ, Cataltepe S, Kumar V, Edwards C, Pace SM (2007). SERPINB11 is a new noninhibitory intracellular serpin. Common single nucleotide polymorphisms in the scaffold impair conformational change.. J Biol Chem.

[36] Lim W, Jeong W, Kim JH, Lee JY, Kim J (2011). Differential expression of alpha 2 macroglobulin in response to dietylstilbestrol and in ovarian carcinomas in chickens.. Reprod Biol Endocrinol.

[37] Giles JR, Shivaprasad HL, Johnson PA (2004). Ovarian tumor expression of an oviductal protein in the hen: a model for human serous ovarian adenocarcinoma.. Gynecol Oncol.

[38] Muramatsu T (1993). Midkine (MK), the product of a retinoic acid responsive gene, and pleiotrophin constitute a new protein family regulating growth and differentiation.. Int J Dev Biol.

[39] Vigny M, Raulais D, Puzenat N, Duprez D, Hartmann MP (1989). Identification of a new heparin-binding protein localized within chick basement membranes.. Eur J Biochem.

[40] Raulais D, Lagente-Chevallier O, Guettet C, Duprez D, Courtois Y (1991). A new heparin binding protein regulated by retinoic acid from chick embryo.. Biochem Biophys Res Commun.

[41] Lai S, Czubayko F, Riegel AT, Wellstein A (1992). Structure of the human heparin-binding growth factor gene pleiotrophin.. Biochem Biophys Res Commun.

[42] Uehara K, Matsubara S, Kadomatsu K, Tsutsui J, Muramatsu T (1992). Genomic structure of human midkine (MK), a retinoic acid-responsive growth/differentiation factor.. J Biochem.

[43] Mitsiadis TA, Salmivirta M, Muramatsu T, Muramatsu H, Rauvala H (1995). Expression of the heparin-binding cytokines, midkine (MK) and HB-GAM (pleiotrophin) is associated with epithelial-mesenchymal interactions during fetal development and organogenesis.. Development.

[44] Sakurai H, Bush KT, Nigam SK (2001). Identification of pleiotrophin as a mesenchymal factor involved in ureteric bud branching morphogenesis.. Development.

[45] Jung CG, Hida H, Nakahira K, Ikenaka K, Kim HJ (2004). Pleiotrophin mRNA is highly expressed in neural stem (progenitor) cells of mouse ventral mesencephalon and the product promotes production of dopaminergic neurons from embryonic stem cell-derived nestin-positive cells.. FASEB J.

[46] Amet LE, Lauri SE, Hienola A, Croll SD, Lu Y (2001). Enhanced hippocampal long-term potentiation in mice lacking heparin-binding growth-associated molecule.. Mol Cell Neurosci.

[47] Hienola A, Pekkanen M, Raulo E, Vanttola P, Rauvala H (2004). HB-GAM inhibits proliferation and enhances differentiation of neural stem cells.. Mol Cell Neurosci.

[48] Muramatsu H, Zou P, Kurosawa N, Ichihara-Tanaka K, Maruyama K (2006). Female infertility in mice deficient in midkine and pleiotrophin, which form a distinct family of growth factors.. Genes Cells.

[49] Brigstock DR, Kim GY, Steffen CL (1996). Pig uterine luminal fluid contains the developmentally regulated neurotrophic factor, pleiotrophin.. J Endocrinol.

[50] Brigstock DR, Kim GY, Steffen CL, Liu A, Vegunta RK (1996). High molecular mass forms of epidermal growth factor in pig uterine secretions.. J Reprod Fertil.

[51] Kohler PO, Grimley PM, O'Malley BW (1968). Protein synthesis: differential stimulation of cell-specific proteins in epithelial cells of chick oviduct.. Science.

[52] Bartel DP (2009). MicroRNAs: target recognition and regulatory functions.. Cell.

[53] Hakim AA, Barry CP, Barnes HJ, Anderson KE, Petitte J (2009). Ovarian adenocarcinomas in the laying hen and women share similar alterations in p53, ras, and HER-2/neu.. Cancer Prev Res (Phila).

[54] Jackson E, Anderson K, Ashwell C, Petitte J, Mozdziak PE (2007). CA125 expression in spontaneous ovarian adenocarcinomas from laying hens.. Gynecol Oncol.

[55] Rodriguez-Burford C, Barnes MN, Berry W, Partridge EE, Grizzle WE (2001). Immunohistochemical expression of molecular markers in an avian model: a potential model for preclinical evaluation of agents for ovarian cancer chemoprevention.. Gynecol Oncol.

[56] Anderson GL, McIntosh M, Wu L, Barnett M, Goodman G (2010). Assessing lead time of selected ovarian cancer biomarkers: a nested case-control study.. J Natl Cancer Inst.

[57] Johnson KA (2009). The standard of perfection: thoughts about the laying hen model of ovarian cancer.. Cancer Prev Res (Phila).

[58] Kim HS, Lim W, Kim YB, Kim MA, Park YS (2011). SERPINB3 in the chicken model of ovarian cancer: a novel biomarker for predicting platinum resistance and survival in patients with epithelial ovarian cancer..

[59] Suzuki K, Oneyama C, Kimura H, Tajima S, Okada M (2011). Down-regulation of the Tumor Suppressor C-terminal Src Kinase (Csk)-binding Protein (Cbp)/PAG1 Is Mediated by Epigenetic Histone Modifications via the Mitogen-activated Protein Kinase (MAPK)/Phosphatidylinositol 3-Kinase (PI3K) Pathway.. J Biol Chem.

[60] Laird PW, Jaenisch R (1994). DNA methylation and cancer.. Hum Mol Genet.

[61] Khalkhali-Ellis Z (2006). Maspin: the new frontier.. Clin Cancer Res.

[62] Jager R, List B, Knabbe C, Souttou B, Raulais D (2002). Serum levels of the angiogenic factor pleiotrophin in relation to disease stage in lung cancer patients.. Br J Cancer.

[63] Souttou B, Juhl H, Hackenbruck J, Rockseisen M, Klomp HJ (1998). Relationship between serum concentrations of the growth factor pleiotrophin and pleiotrophin-positive tumors.. J Natl Cancer Inst.

[64] Klomp HJ, Zernial O, Flachmann S, Wellstein A, Juhl H (2002). Significance of the expression of the growth factor pleiotrophin in pancreatic cancer patients.. Clin Cancer Res.

[65] Soulie P, Heroult M, Bernard-Pierrot I, Caruelle D, Oglobine J (2004). Correlation of elevated plasma levels of two structurally related growth factors, heparin affin regulatory peptide and midkine, in advanced solid tumor patients.. Cancer Detect Prev.

[66] Zhang N, Zhong R, Wang ZY, Deuel TF (1997). Human breast cancer growth inhibited in vivo by a dominant negative pleiotrophin mutant.. J Biol Chem.

[67] Choudhuri R, Zhang HT, Donnini S, Ziche M, Bicknell R (1997). An angiogenic role for the neurokines midkine and pleiotrophin in tumorigenesis.. Cancer Res.

[68] Lee SI, Lee WK, Shin JH, Han BK, Moon S (2009). Sexually dimorphic gene expression in the chick brain before gonadal differentiation.. Poult Sci.

[69] Sanders MM, McKnight GS (1988). Positive and negative regulatory elements control the steroid-responsive ovalbumin promoter.. Biochemistry.

[70] Seo HW, Park JY, Lee HC, Kim D, Song YS (2009). Physiological Effects of Diethylstilbestrol Exposure on the Development of the Chicken Oviduct.. J Anim Sci & Technol.

[71] Kohler PO, Grimley PM, O'Malley BW (1969). Estrogen-induced cytodifferentiation of the ovalbumin-secreting glands of the chick oviduct.. J Cell Biol.

[72] McKnight GS (1978). The induction of ovalbumin and conalbumin mRNA by estrogen and progesterone in chick oviduct explant cultures.. Cell.

[73] Song G, Bazer FW, Spencer TE (2007). Pregnancy and interferon tau regulate RSAD2 and IFIH1 expression in the ovine uterus.. Reproduction.

[74] Livak KJ, Schmittgen TD (2001). Analysis of relative gene expression data using real-time quantitative PCR and the 2(−Delta Delta C(T)) Method.. Methods.

[75] Song G, Spencer TE, Bazer FW (2006). Progesterone and interferon-tau regulate cystatin C in the endometrium.. Endocrinology.

[76] Drummond AJ, Ashton B, Buxton S, Cheung M, Cooper A (2010). http://www.geneious.com.

